# Biodegradable hollowed mesoporous SeO_2_ nanoplatform loaded with indocyanine green for simultaneous NIR II fluorescence imaging and synergistic breast carcinoma therapy

**DOI:** 10.3389/fbioe.2023.1151148

**Published:** 2023-03-16

**Authors:** Tingwei Peng, Qing Liu, Hui Song, Conghui Zhang, Xue Wang, Ping Ru, Tianzhao Xu, Xinghui Liu

**Affiliations:** ^1^ Postgraduate Training Base at Shanghai Gongli Hospital, Ningxia Medical University, Shanghai, China; ^2^ Department of Clinical Laboratory, Shanghai Gongli Hospital, The Second Military Medical University, Shanghai, China; ^3^ Department of Obstetrics, Shanghai East Hospital, School of Medicine, Tongji University, Shanghai, China; ^4^ Hospital Department, Shanghai University of Medicine and Health Sciences Affiliated to Zhoupu Hospital, Shanghai, China

**Keywords:** hollowed mesoporous SeO_2_, ICG precise delivery, NIR II fluorescent imaging, photothermal therapy, ROS mediated oxidative therapy

## Abstract

Contrast agents in the second window of the near-infrared region (NIR II, 1000–1700 nm) have several advantages and indocyanine green (ICG), which emits NIR II fluorescence, is clinically approved and its use has been widely investigated for *in vivo* imaging, specifically for delineating tumor outlines; however, insufficient tumor targeting and rapid physiological metabolism of free ICG has substantially impeded its further clinical application. Here, we constructed novel hollowed mesoporous selenium oxide nanocarriers for precise ICG delivery. After surface modification with the active tumor targeting amino acid motif, RGD (hmSeO_2_@ICG-RGD), the nanocarriers were preferentially targeted toward tumor cells and subsequently degraded for ICG and Se-based nanogranule release under tumor tissue extracellular pH conditions (pH 6.5). The released ICG acted as an NIR II contrast agent, highlighting tumor tissue, after intravenous administration of hmSeO_2_@ICG-RGD into mammary tumor-bearing mice. Importantly, the photothermal effect of ICG improved reactive oxygen species production from SeO_2_ nanogranules, inducing oxidative therapy. The synergistic therapeutic effects of hyperthermia and increased oxidative stress on 808 nm laser exposure induced significant tumor cell killing. Thus, our nanoplatform can generate a high-performance diagnostic and therapeutic nanoagent that facilitates *in vivo* tumor outline discrimination and tumor ablation.

## 1 Introduction

Optical imaging using the second biological transparency window of the near-infrared region (NIR II, 1000–1700 nm) has considerable promise for fluorescent imaging because it generates low levels of light scattering in tissue and a higher penetration depth than imaging using the first window (NIR I, 700–900 nm) (Li et al., 2018a; Fan et al., 2018; Jiang et al., 2018; Wang et al., 2019a). Recently, indocyanine green (ICG), an FDA-approved clinical imaging contrast agent, was found to emit NIR II fluorescence (1000–1500 nm) under 808 nm laser irradiation using an InGaAs camera (Starosolski, et al., 2017; Bhavane et al., 2018; Carr et al., 2018; Wang, et al., 2019b; Yang et al., 2022); However, ICG has several limitations for *in vivo* imaging. First, ICG is generally rapidly cleared from the blood circulatory system (blood half-life, approximately 3 min) (Dorr and Pollack, 1989; Saxena et al., 2003). Second, due to its amphiphilic properties, ICG non-covalently interacts with various proteins, including lipoproteins and human serum albumin, and forms aggregates *via* physical mechanisms (Patonay et al., 2004; Russell et al., 2022). Third, ICG lacks tumor targeting capacity, resulting in low signal to background ratios (Zheng et al., 2012; Keating et al., 2016). Accordingly, ICG requires assistance from an additional drug delivery system for further application in tumor imaging.

Generally, an optimal nanocarrier should possess the following properties: 1) High stability and outstanding dispersion, to allow good blood circulation behavior with prolonged half-life in blood and increased cargo bioavailability; 2) Superior biocompatibility and excellent biodegradability, to guarantee high *in vivo* biosafety; 3) High cargo loading capacity, which can reduce the required dosage and *in vivo* administration frequency; 4) Precisely targeted drug delivery ability, to realize bioavailability and avoid off-target effects on healthy tissue; 5) Controllable drug release capacity, to increase drug bioavailability and reduce side effects (Lopez-Davila et al., 2012; Kang et al., 2015; Cai et al., 2020; Cheng et al., 2021). Unfortunately, it is difficult for traditional nanomaterials to meet all of these requirements. For example, organic nanocarriers often have flexible designability and can be fabricated according to requirements for various application, while they have poor thermal and micelle stability (Ma et al., 2015). In contrast, inorganic nanocarriers present high stability, while their biodegradability is an ongoing problem. Further, the risks of long-term toxicity *in vivo* need to be carefully addressed for some inorganic nanocarriers, which severely impedes their clinical applications (Li, et al., 2018b; Yang et al., 2019).

As a non-metal chemical element, selenium (Se) is primarily detected in sedimentary rocks and soils and is an essential trace element required by humans. Immobilized Se becomes bioavailable through soil weathering or reduction by microorganisms (Southwell-Keely et al., 1974; Sarkar et al., 2015). Importantly, dietary supplementation with Se has various beneficial effects on human health (Rayman, 2000; Zhang et al., 2001; Hosnedlova et al., 2017; Hosnedlova et al., 2018), and Se has been suggested as a promising candidate for preventing and eradicating tumor progression (Sanmartin et al., 2012). Recently, Se-containing nanomaterials have attracted considerable attention in the field of tumor therapy (Bjornstedt and Fernandes, 2010; Fang et al., 2018; Song et al., 2018; Zhao et al., 2018). In addition to their use as antitumor drug nanocarriers, these nanomaterials also exhibit antitumor activities, based on the unique biological functions of Se (Fernandes and Gandin, 2015; He et al., 2018; Pan et al., 2019). During selenocysteine formation, Se is incorporated into numerous selenoproteins and plays a vital role in regulating the physiological redox balance in humans (Razaghi et al., 2021). At low nutritional levels, Se displays antioxidant properties; however, it exerts pro-oxidant activity at elevated doses, resulting in production of reactive oxygen species (ROS) (Feng et al., 2014; Li and Xu, 2020). Relative to healthy cells, both glycolysis and the pentose phosphate cycle exacerbate the high levels of ROS and redundant agents in tumor cells (Wang et al., 2012). There is increasing evidence that the high innate ROS generation by tumor cells makes them particularly vulnerable to additional ROS production, which can be harnessed as ROS-mediated oxidative therapy (Lv et al., 2021). Accordingly, Se-containing nanocarriers that generate ROS have potential for use as antitumor therapeutics (Li et al., 2015; Xia et al., 2020). The anti-tumor properties of Se compounds have been proven in clinical trials, where Se supplementation was most effective against colorectal, prostate, and lung cancers.

**FIGURE 1:**
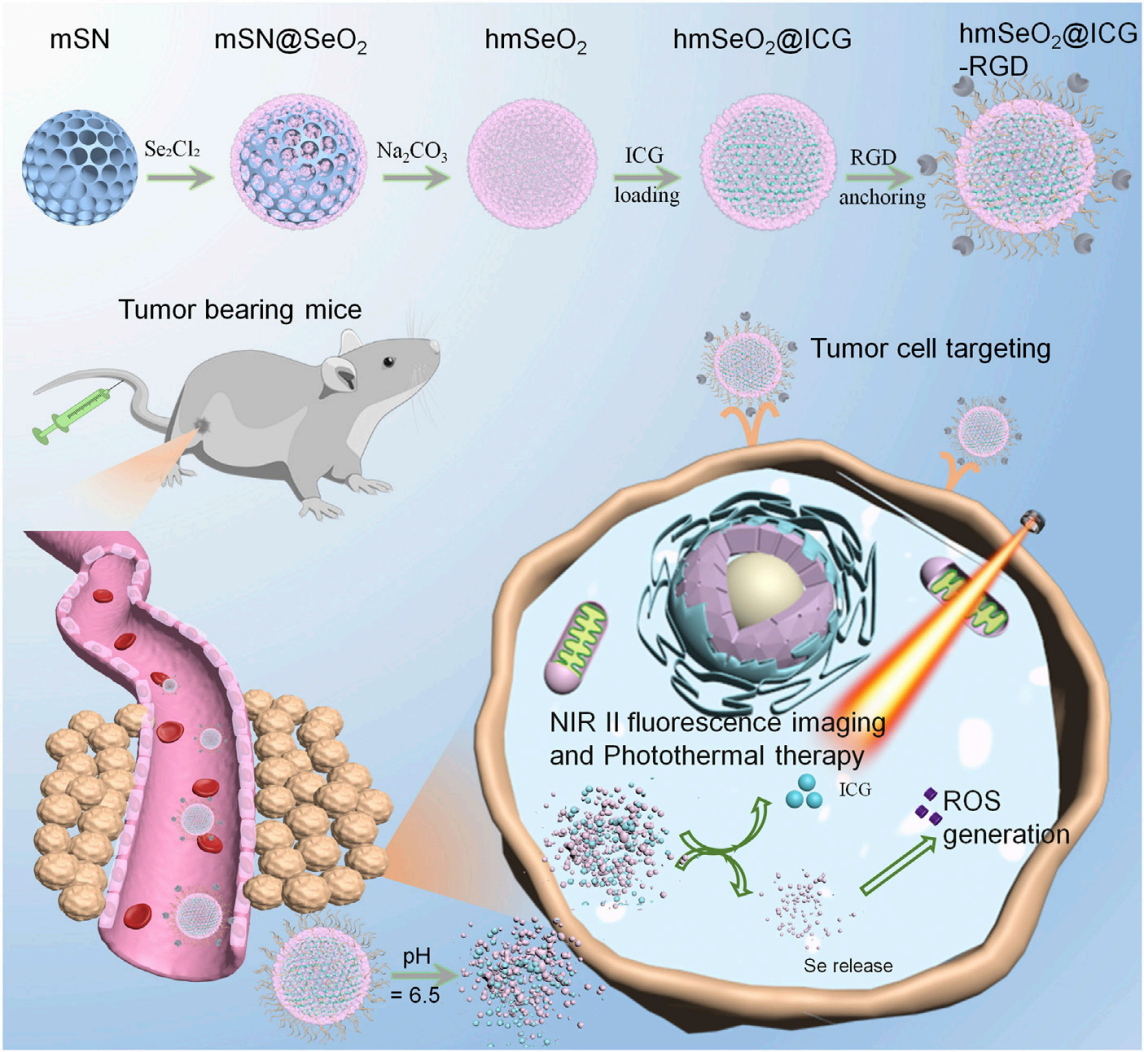
Schematic illustration of step-wise hmSeO_2_@ICG-RGD synthesis for NIR II fluorescence imaging and synergistic tumor eradiation *via* photothermal therapy and ROS mediated oxidative therapy.

Here, we describe fabrication of a multi-functional nanoplatform, comprising hollowed mesoporous Selenium dioxide (hmSeO_2_) nanospheres, using hard-template strategy. The hollowed nanostructures and mesoporous channels enabled the efficient incorporation of ICG molecules (hmSeO_2_@ICG), while surface modification with tumor targeting RGD peptides (hmSeO_2_@ICG-RGD) facilitated targeted delivery of ICG to tumor tissues *via* systemic administration. These Se-based nanocarriers underwent biodegradation in the acidic extra-tumor microenvironment, where they simultaneously released both SeO_2_ nanogranules and ICG molecules. ICG acted as an NIR II contrast agent which successfully discriminated tumor outlines. SeO_2_ nanoparticles generated large amounts of ROS, particularly under hyperthermia stimulation *via* 808 nm laser irradiation (Figure 1). The photothermal effects of ICG and disrupted ROS cytoplasm levels induced efficient antitumor effects, both *in vivo* and *in vitro*. These results demonstrate that biodegradable SeO_2_ nanocarriers represent potentially useful reagents for use in tumor diagnostics, with simultaneous potent cancer inhibition properties.

## 2 Materials and methods

### 2.1 Mesoporous silica preparation

Traditional mesoporous silica nanospheres were fabricated using a general bi-phase method, with hexadecyltrimethylammonium (CTAB, 1.5 g) dispersed in 60 ml deionized water, followed by addition of 0.72 ml triethanolamine (25%) aqueous solution as a reductant, after 30 min stirring at 60°C in a dimethyl silicone oil bath, containing 20 mL tetraethyl orthosilicate and cyclo-hexane mixture (volume ratio, 1:4). The whole system was maintained in an oil bath for 48 h. Thereafter, the generated silica nanospheres were alternately washed several times with deionized water and anhydrous ethanol by centrifugation (10 min, 13,000 rpm). Finally, the mesoporous nanospheres were dispersed in acetone (50 ml) and continuously refluxed at 60°C overnight to remove residual CTAB. The final mesoporous products were washed several times with water and ethanol alternately, then re-dispersed in 10 ml deionized water and stored at 4°C.

### 2.2 HmSeO_2_ fabrication

For hmSeO_2_@ICG synthesis, 100 mg of mesoporous silica nanospheres and 0.1 g of Se_2_Cl_2_ were concurrently added in 60 ml deionized water and then poured into a 100 ml single-neck bottle and stirred at 90°C in a dimethyl silicone oil bath for 30 min. Then, 0.10 g of hexamethylenetetramine was carefully added to the mixture of silica and Se precursor, followed by vigorous stirring at 90°C in a dimethyl silicone oil bath for >3 h. Final samples, comprising core-shell nanostructures (mSN@SeO_2_), were subjected to three more centrifugal washes in deionized water (10 min, 5000 rpm) followed by overnight freeze drying. Finally, mesoporous silica-based hard templates were removed using 60 ml Na_2_CO_3_ aqueous solution (0.1 M). After stirring at 75°C overnight in in a dimethyl silicone oil bath and three more centrifugal washes in deionized water (10 min, 5000 rpm), as-made samples were freeze dried overnight to obtain hmSeO_2_ nanospheres, which were re-dispersed in deionized water.

### 2.3 hmSeO_2_@ICG preparation

ICG powder (1 mg) was added into anhydrous ethanol (2.0 ml). Then, 25 mg of the hmSeO_2_, prepared as described above, was dispersed in ICG solution and the ICG-hmSeO_2_ mixture continuously stirred at room temperature (RT) for >48 h. ICG molecules were successfully loaded in the stacking mesopores of SeO_2_ nanogranules, as well as in the hollow cavity of hmSeO_2_. The as-prepared ICG-loaded hmSeO_2_ nanospheres (hmSeO_2_@ICG) were centrifugally washed >3 times in deionized water (10 min, 5000 rpm).

### 2.4 hmSeO_2_@ICG-RGD synthesis

hmSeO_2_@ICG (100 mg) and DSPE-PEG_2000_-NH_2_ powder (25 mg) were concurrently added into 10 mL deionized water and amino group-anchored hmSeO_2_@ICG (hmSeO_2_@ICG-NH_2_) obtained by gently stirring for 12 h at RT. After removal of residual DSPE-PEG_2000_-NH_2_ by centrifugation (20 min, 15000 rpm), as-prepared hmSeO_2_@ICG-NH_2_ was collected and re-dispersed in 10 ml deionized water. Next, hmSeO_2_@ICG-RGD was synthesized. First, 6 mg RGD power was dissolved in 5 ml, 0.1 M 2-(N-morpholino) ethanesulfonic acid buffer (pH, 5.5). Then, EDC (N-(3-dimethylaminopropyl)-N′-ethylcarbodiimide hydrochloride) power (20 mg) and NHS N-hydroxysuccinimide powder (20 mg) were concurrently added into the mixture, with continuous stirring for 2 h at RT. Subsequently, 60 mg hmSeO_2_@ICG was added to the system and continuous stirring maintained for 12 h. RGD-modified hmSeO_2_@ICG was centrifugally washed using deionized water (10 min, 5000 rpm) and the final hmSeO_2_@ICG-RGD sample re-dispersed in 20 ml deionized water.

### 2.5 ROS detection in cytoplasm

ROS generated by hmSeO_2_@ICG-RGD in cytoplasm was visualized by confocal laser scanning microscopy (CLSM). Briefly, 4T1 breast tumor cells were first seeded into 6-well plates (density, 1 × 10^5^/well) and cultured for 24 h in 1640 medium containing 10% fetal bovine serum. After treatment with 100 μg/ml hmSeO_2_@ICG-RGD for 12 h, an 808 nm laser was employed for continuous irradiation (5 min, 1 W/cm^2^). Control 4T1 cells co-cultured with hmSeO_2_@ICG-RGD were prepared using the same procedure as for hmSeO_2_@ICG-RGD + laser-treated cells. Further, ROS levels were estimated by 2′,7′-dichlorodihydrofluorescein diacetate (DCHF-DA) staining and observed under CLSM.

### 2.6 NIR II fluorescent imaging for tumor targeting evaluation

Studies involving human participants were reviewed and approved by The Ethical Committee of Ningxia Medical University. All animal studies were approved by The Second Military Medical University, and performed in accordance with the corresponding relevant guidelines (Shanghai, China). Female BALB/c nude mice (6-week-old) were purchased from Laboratory Animal Co., Ltd. (Shanghai Laboratory Animal Center). Subcutaneous breast carcinoma-bearing nude mice were generated by 10 days of transcutaneous injection of 4T1 carcinoma cells (1 × 10^6^ dispersed in 150 μl PBS) in the right hind-limb. Tumors were allowed to grow to a volume of approximately 200 mm^3^, then free ICG and hmSeO_2_@ICG-RGD were intravenously administered into the carcinoma-bearing mice (n = 4 per group). Thereafter, all mice in the two groups were imaged using a home-made NIR fluorescent imaging system (NIR-OPTICS Series III 900/1700) at various times post-injection. Major organs (heart, liver, spleen, lung, kidney) and resected tumors were collected from the two groups of mice 72 h post-injection and *ex vivo* NIR II fluorescent images obtained using our imaging system. Thermographic imaging was subsequently performed for *in vivo* tumor targeting evaluation. Tumor-bearing BALB/c nude mice were subjected to tail vein injection of free ICG and hmSeO_2_@ICG-RGD (n = 4 per group). Then, an 808 nm laser was employed for continuous illumination (5 min, 1 W/cm^2^) of tumor sites at various times post-injection. Typically, *in vivo* photothermal images were obtained using a thermal camera (FOTRIC 225) to detect the infrared region.

### 2.7 *In vivo* tumor ablation efficiency

Breast carcinoma-bearing Balb/c nude mice were first divided into four groups (n = 4 mice per group), as follows: 1) PBS, 2) ICG, 3) hmSeO_2_@ICG-RGD, 4) hmSeO_2_@ICG-RGD + laser. Mice were subjected to intravenous administration twice (days 0 and 8) and tumor inhibition evaluated. Continuous 808 nm laser illumination (5 min, 1 W/cm^2^) was conducted 24 h post-injection. The Se-based nanoplatform was used at approximately 8 mg/kg in groups 3 and 4, while, mice in the free ICG group received an equivalent ICG dose as those in the hmSeO_2_@ICG-RGD group. Digital photographs of all mice were taken on days 0, 7, and 15. Tumor size (width and length) was measured on various days using a digital caliper and tumor volumes in the four groups calculated using the formula (width × width × length)/2. Simultaneously, mouse body weights were measured at various time points during tumor ablation. Finally, after administration of PBS, ICG, hmSeO_2_@ICG-RGD, or hmSeO_2_@ICG-RGD + laser for 1 week, tumors were dissected and sectioned (approximately 10 μm thickness). Hematoxylin and eosin (H&E) and TUNNEL staining were conducted immediately. Further, five major organs (heart, liver, spleen, lung, and kidney) were assessed by H&E staining after PBS, ICG, hmSeO_2_@ICG-RGD, or hmSeO_2_@ICG-RGD + laser treatment at the end of the tumor ablation period.

## 3 Results and discussion

### 3.1 Stepwise fabrication of hmSeO_2_@ICG-RGD

**FIGURE 2:**
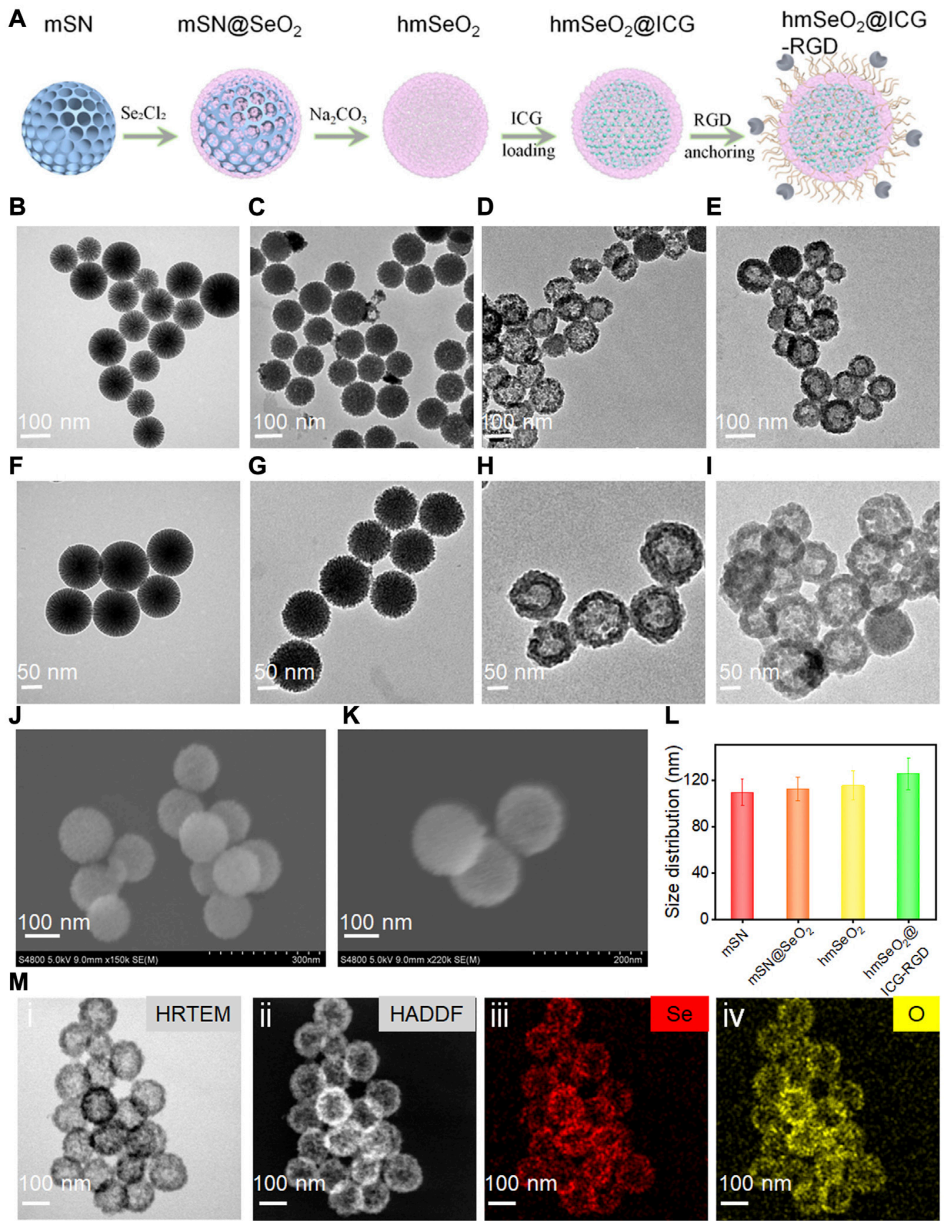
**(A)** Schematic illustrating step-by-step hmSeO_2_@ICG-RGD construction. **(B–I)** Large-scale and magnified TEM images of mSN **(B, F)**, mSN@SeO_2_ **(C, G)**, hmSeO_2_ **(D, H)**, and hmSeO_2_@ICG-RGD **(E, I)**. SEM images of hmSeO_2_ and hmSeO_2_@ICG-RGD **(J, K)**. **(L)** Size distributions of mSN, mSN@SeO_2_, hmSeO_2_, and hmSeO_2_@ICG-RGD. **(M-i)** HRTEM, **(ii)** HADDF, and EDS mapping images of **(iii)** Se, and **(iv)** O.

The specific detailed steps involved in hmSeO_2_@ICG-RGD fabrication are illustrated in Figure 2A. First, hard-template, mesoporous silica nanospheres (mSN) were synthesized *via* a versatile bi-phase method, as previously reported,^7^ and visualized by transmission electron microscopy (TEM) (Figures 2B, F). Template nanospheres were monodispersed and uniform, with mean diameter approximately 109 nm. Second, SeO_2_ nanogranules with stacked mesopores were directly nucleated on the surface of the mSN using the reductant, methenamine (Figures 2C, G). To generate a large specific surface area for drug delivery, the inner template was then removed using 0.1 M sodium hydroxide. Hollowed mesoporous SeO_2_ nanospheres (hmSeO_2_) were clearly obtained and their diameter was slightly increased (approximately 115 nm) (Figures 2D, H, L). Third, ICG molecules were added into the hollowed cavities and stacking mesopores. Finally, to achieve good biocompatibility and allow further modification, DSPE-PEG_2000_-NH_2_ was conjugated on the exterior of as-prepared hmSeO_2_. The breast tumor-specific targeting peptide, RGD, was anchored on the hmSeO_2_ surface by amino group modification using a general EDC/NHS reaction (hmSeO_2_@ICG-RGD). The morphology of this nanoplatform was further examined by TEM, which revealed that the hollowed nanostructures remained completely intact after ICG encapsulation and RGD conjugation, with hydrated particle size approximately 125 nm (Figures 2E, I, L), demonstrating the reliability of our strategy for novel nanoplatform construction for ICG delivery and precise tumor recognition. Further, both monodispersed hmSeO_2_ and hmSeO_2_@ICG-RGD with radial pore nanostructures were clearly visualized by scanning electron microscopy (SEM) (Figures 2J, K). In addition, high angle angular dark field-scanning transmission electron microscope and high-resolution TEM images revealed the hollowed morphology and homogeneous size of our novel nanoplatform (Figure 2M). The nanoplatform was further analyzed using energy-dispersive X-ray spectroscopy mapping images, which demonstrated that the hybrid nanocomplex comprised Se and O atoms (Figure 2M).

### 3.2 Photothermal and biodegradable evaluation of hmSeO_2_@ICG-RGD

**FIGURE 3:**
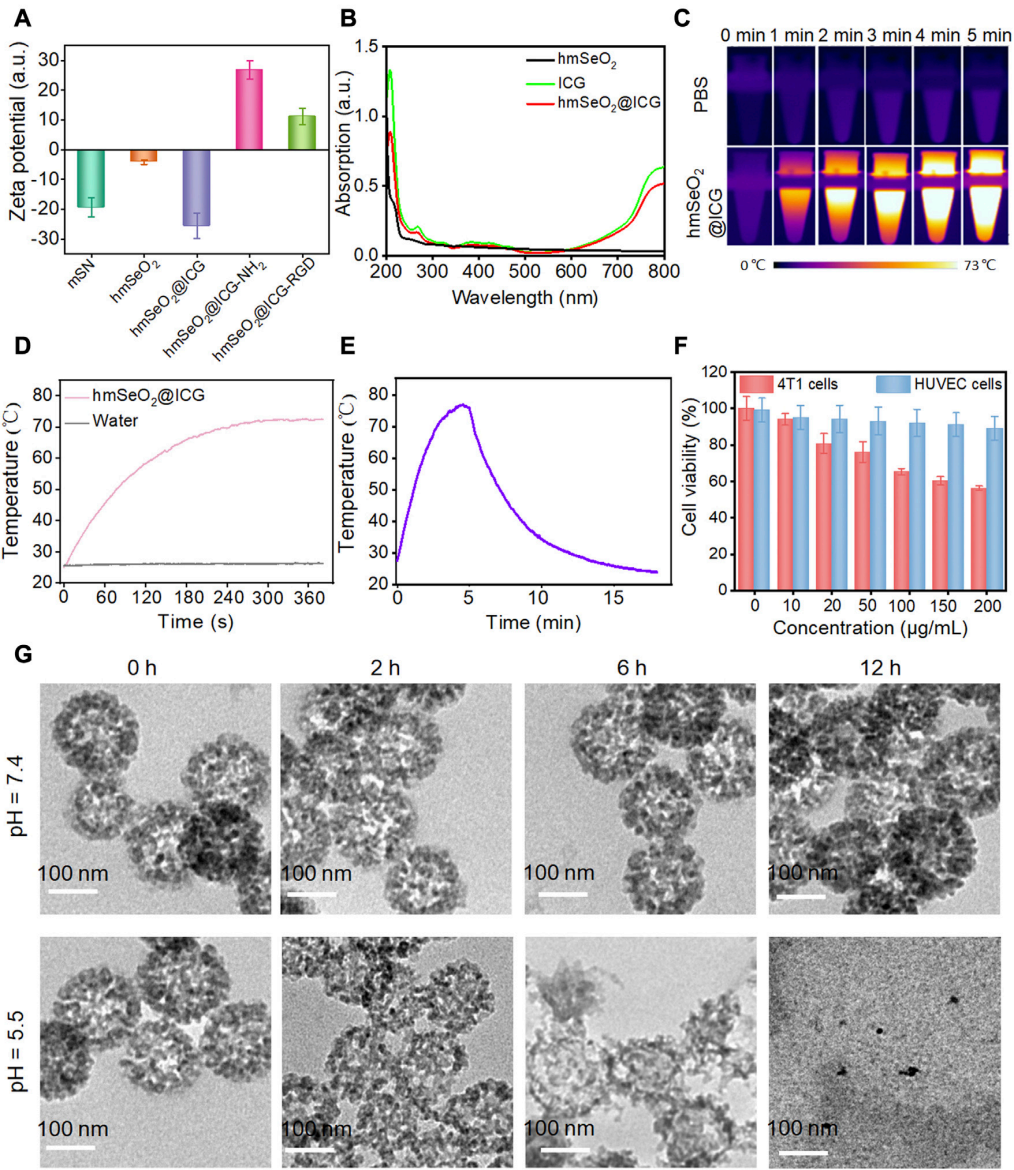
**(A)** Zeta potential values of mSN, hmSeO_2_, hmSeO_2_@ICG, hmSeO_2_@ICG-NH_2_, and hmSeO_2_@ICG-RGD. **(B)** UV-vis spectra of hmSeO_2_, ICG, and hmSeO_2_@ICG. **(C)** IR thermal images of hmSeO_2_@ICG-RGD and PBS after 808 nm laser exposure for various durations. **(D)** Temperature *vs*. laser irradiation time curves of hmSeO_2_@ICG-RGD and PBS groups. **(E)** Photothermal effect of hmSeO_2_@ICG on 808 nm laser irradiation for 5 min, after which the laser was turned off. **(F)** Viability of 4T1 and HUVEC cells after incubation with hmSeO_2_@ICG at various concentrations for 12 h **(G)** TEM images of hmSeO_2_@ICG after dispersal in PBS or pH = 6.5 buffer for various periods of time.

To assess whether ICG loading and RGD anchoring were successful, we next conducted zeta potential and UV-vis spectrum studies. As shown in Figure 3A, after ICG encapsulation, more negative charges were detected on the nanoparticles, consistent with a previous report (Tan et al., 2022). Interestingly, a distinct charge reversal was discovered after amino group modification, with a drastic charge decrease in hmSeO_2_@ICG-RGD. All ICG characteristic peaks were also present in the hmSeO_2_@ICG-RGD absorbance spectrum (Figure 3B). Together, these findings indicate successful ICG delivery and surface conjugation of RGD. By absorbance spectrum of ICG, the mass loading capacity of ICG in the hollow capacity and stacked mesopores was apprised as −18.2 wt%. In addition, we also estimated the photothermal efficacy of our nanoplatform using 808 nm laser irradiation. As shown in Figure 3C, infrared thermal images of 150 μg/ml hmSeO_2_@ICG-RGD solution revealed a pronounced temperature increase, reaching a maximum of approximately 73.0°C after 5 min laser illumination (1.0 W/cm^2^). No temperature increase was detected in the PBS group, even after 5 min laser irradiation. Additionally, hmSeO_2_@ICG-RGD temperature rose with longer laser irradiation time, from 26.3°C to 72.5°C, while negligible temperature increase was detected in the PBS group. The maximum temperature gradually reduced back to the initial level after the laser was powered off for >5 min and the photothermal conversion efficiency (η) of hmSeO_2_@ICG-RGD was determined as high as 39.87% (Supplementary Figure S1). All these data provided preliminarily evidence of the laser-dependent performance of our nanoplatform (Figures 3D, E).

hmSeO_2_@ICG-RGD biodegradation behavior was investigated in buffer mimicking the pH values in the extracellular environment of tumor cells. TEM images showed that, after 6 h incubation in a pH 6.5 solution, the SeO_2_-based nanoplatform initially underwent biodegradation, with surface and bulk erosion. The hollowed hmSeO_2_ nanostructures disintegrated into small nanogranules (approximately 7 nm) after 12 h incubation, which may facilitate *in vivo* renal clearance of hollowed Se-based nanospheres. In contrast, negligible structural transformation was detected in the PBS-treated group, even following an extended period of incubation (Figure 3G). These phenomena may be attributable to the vulnerability created by the stacking force between Se-based nanogranules during hmSeO_2_ fabrication. Besides, approximately 80% of ICG was release in pH = 5.5 group at this timepoint, evidently, only 20% of ICG was found in pH = 7.4 group, further verifying the effectively biodegradable behavior of our mesoporous nanocarriers (Supplementary Figure S2). The biodegradable characteristics of our nanoplatform may facilitate precise release of loaded cargos into tumor tissues and effective internal permeation of tumors. Importantly, hmSeO_2_@ICG exhibited remarkably higher cytotoxicity against the breast tumor cell line (4T1), with only 56.12% living cells detected after co-culture with 200 μg/ml Se-based nanocarriers for 12 h (Figure 3F). In sharp contrast, >90% of normal cells (HUVEC) survived even after treatment with the highest nanocarrier concentration, demonstrating that hmSeO_2_ exhibits tumor-microenvironment responsive biodegradation and ROS generation (Figure 3F).

### 3.3 Cellular uptake, ROS generation, and cell killing effects

**FIGURE 4:**
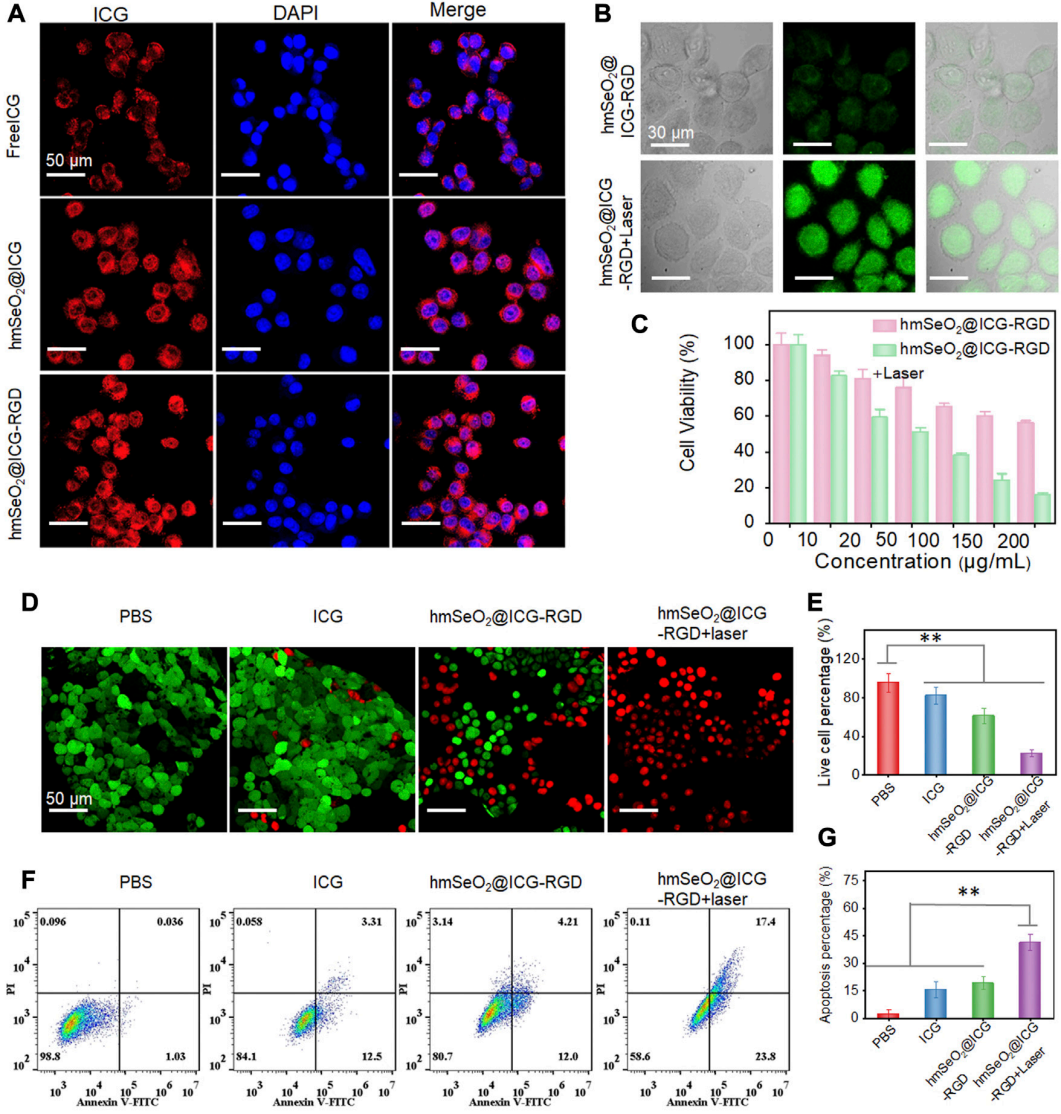
**(A)** CLSM images of 4T1 cells after incubation with free ICG, hmSeO_2_@ICG and hmSeO_2_@ICG-RGD for 12 h. **(B)** Levels of ROS generation in the cytoplasm of 4T1 cells after treatment with hmSeO_2_@ICG-RGD and hmSeO_2_@ICG-RGD + laser. **(C)** Assessment of cytotoxicity to 4T1 cells of treatment with hmSeO_2_@ICG-RGD and hmSeO_2_@ICG-RGD + laser at various concentrations. **(D)** CLSM images of 4T1 cells after various treatments followed by Calcein-AM/PI staining. **(E)** Quantitative analysis of percentages of live 4T1 cells after various treatments. **(F)** Flow cytometry analysis of 4T1 cells after various treatments followed by staining with Annexin V/PI. **(G)** Quantitative analysis of cell apoptosis rates in the four treatment groups. **p < 0.01.

Generally, cellular uptake efficacy is a prerequisite for *in vivo* application of anti-tumor agents. Accordingly, the internalization of free ICG, hmSeO_2_@ICG, and hmSeO_2_@ICG-RGD in 4T1 cells was assessed by monitoring ICG red fluorescence using CLSM. After 12 h incubation, hmSeO_2_@ICG-RGD demonstrated the highest red fluorescence intensity, with fluorescence generated by free ICG lower than that of the nanocarriers group (hmSeO_2_@ICG) (Figure 4A). Particularly, in sharp contrast with free ICG incubated cells, about 5 times of fluorescence intensity is found in hmSeO_2_@ICG-RGD treated cells (Supplementary Figure S3). These results indicate precise tumor cell targeting and effective ICG delivery performance by our novel hmSeO_2_@ICG-RGD nanoplatform. Given the efficient endocytosis of hmSeO_2_@ICG-RGD, we next evaluated the ROS generation capability of Se based nanoparticles using a commercial indicator, DCFH-DA. Relatively low green fluorescence levels, due to ROS production by the Se-based nanocarriers, were observed in the hmSeO_2_@ICG-RGD group. Importantly, the green signal intensity was enhanced 4.2 times after 808 nm laser irradiation (1 W/cm^2^, 5 min), suggesting improvement of ICG catalytic activity due to hyperthermia (Figure 4B; Supplementary Figure S4).

Next, 4T1 cell survival rates in the hmSeO_2_@ICG-RGD and hmSeO_2_@ICG-RGD + laser groups were evaluated using a CCK-8 kit. In the beginning, over 85% tumor cells still survived even the laser irradiation time prolonged to 10 min at the power density of 1 W/cm^2^, preliminarily demonstrating the biosafety of the NIR laser (Supplementary Figure S5). After incubation with hmSeO_2_@ICG-RGD for 12 h, cells were continuously illuminated using an 808 nm laser (5 min, 1 W/cm^2^). The live cell rate decreased to 15.72% when the hmSeO_2_@ICG-RGD concentration reached 200 μg/ml, which was markedly lower than that in cells treated with nanoplatform only (Figure 4C). This can be primarily ascribed to photothermal effects inducing improvement of ROS generation, resulting in massive cell killing. Further, hmSeO_2_@ICG-RGD + laser (100 μg/ml) induced distinct cytotoxicity, as visualized by CLSM after Calcein-AM/propidium iodide (PI) staining for discrimination of live and dead cells. No significant cell death was detected in cells treated with PBS or free ICG, confirmed by strong Calcein-AM green fluorescence intensity. By contrast, hmSeO_2_@ICG-RGD induced significant cell death (visualized as PI-mediated red fluorescence), demonstrating a ROS mediated oxidation killing effect that induced death of a proportion of cells (Figure 4D). Notably, the strongest red fluorescence intensity was observed in cells treated with hmSeO_2_@ICG-RGD accompanied by 808 nm laser irradiation (1 W/cm^2^, 5 min), resulting in a significantly lower cell survival rate (22.51%) (Figures 4D, E). Additionally, the apoptosis/necrosis rate of cells treated with hmSeO_2_@ICG-RGD + laser was evaluated by flow cytometry following Annexin V/PI staining. Relative to cells treated with PBS, free ICG, or hmSeO_2_@ICG-RGD, the highest apoptosis/necrosis rate was detected in hmSeO_2_@ICG-RGD treated cells after 808 nm laser illumination (Figures 4F, G). This finding is consistent with the results of analyses of cell death and comparisons of live/dead cells (Figures 4C, E), and indicates a synergistic tumor cell inhibition effect between photothermal therapy (PTT) and ROS-induced oxidation therapy.

### 3.4 *In vivo* NIR II fluorescence imaging assessment

**FIGURE 5:**
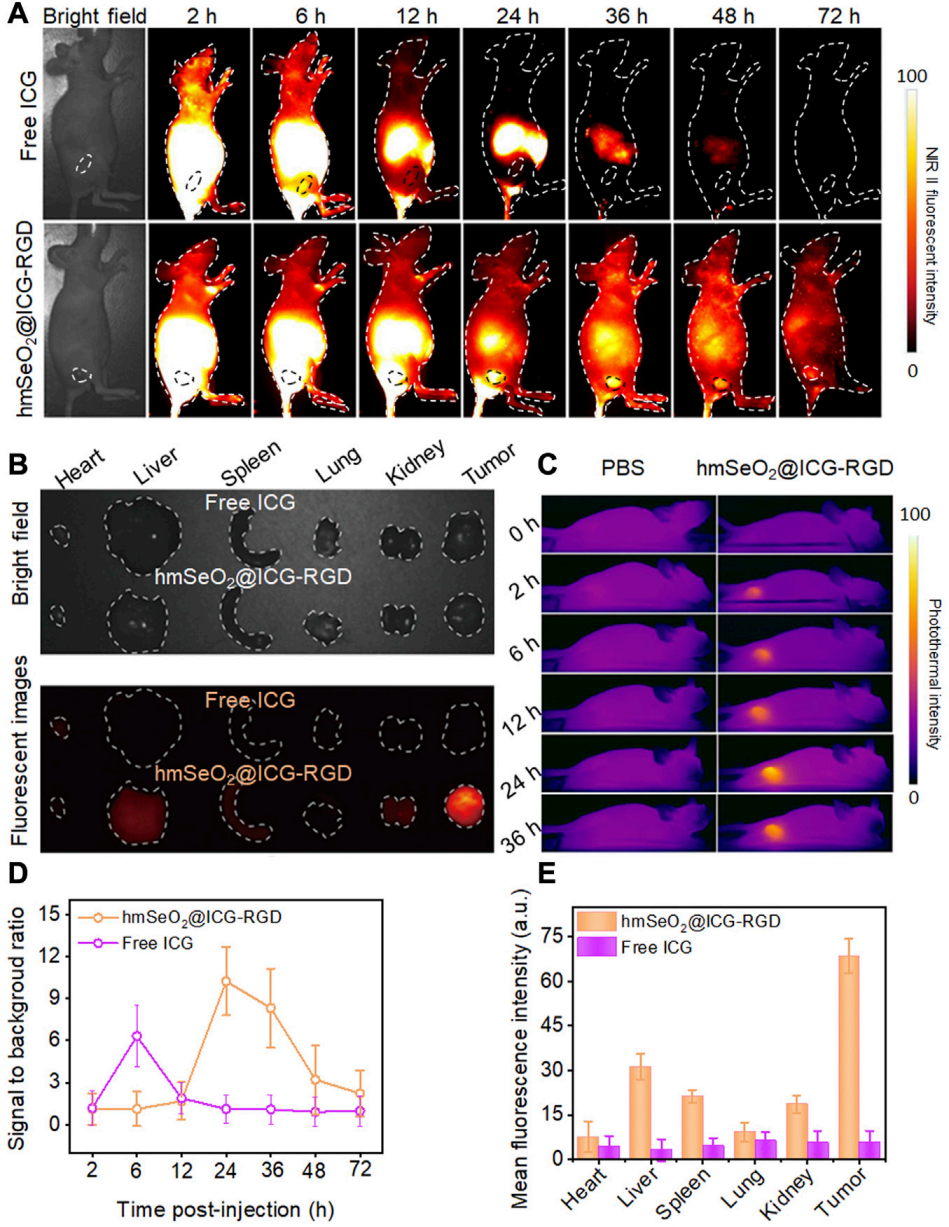
**(A)** NIR II fluorescent images of breast carcinoma-bearing nude mice after tail vein injection of hmSeO_2_@ICG-RGD and ICG at different time-points. **(B)** *Ex vivo* bright field and fluorescent imaging of major organs (heart, liver, spleen, lung, kidney) and tumors dissected from mice after intravenous injection with hmSeO_2_@ICG-RGD and ICG for 72 h, respectively. **(C)** *In vivo* photothermal images of 4T1 tumor-bearing mice after treatment with free ICG and hmSeO_2_@ICG-RGD for various periods of time. **(D)** Signal to background ratios in 4T1 tumor-bearing mice after treatment with free ICG or hmSeO_2_@ICG-RGD for various periods of time. **(E)** Quantitative mean fluorescent intensity values determined from *ex vivo* fluorescent images.

Before conducting *in vivo* tumor eradication experiments, the timepoint at which maximum tumor accumulation of nanocarriers occurred was determined using female nude mice bearing subcutaneous mammary tumors after tail vein injection of free ICG or hmSeO_2_@ICG-RGD. *In vivo* NIR II fluorescent images (1000 nm long-pass filter) were obtained under illumination with an 808 nm laser (100 mW/cm^2^) at various time-points post-injection. As shown in Figure 5A, the fluorescence intensity in tumor tissues continuously increased over the time, due to the activity of the tumor-targeting RGD-modified Se-based nanoparticles. Although the NIR II signal intensity reached a peak at 12 h, the signal to background (tumor to adjacent normal tissue) ratio significantly increased from 1.89 to 10.23 at 24 h post-injection (Figures 5A, D). Therefore, we chose to conduct laser irradiation for PTT at 24 h post-injection, where there was maximum tumor accumulation level without damage of surrounding normal tissue. Surprisingly, the NIR II fluorescence signals from tumor regions continued for an extended period, highlighting tumor outlines even at 3 days post-injection, while in the ICG group, tumor delineation failed as early as 6 h post-injection, with the highest signal to background ratio (6.31) and negligible fluorescence signal detected after this time point, likely attributable to insufficient tumor targeting and short half-life of ICG in blood (Figures 5A, D). Simultaneously, tumor tissues were collected for the corresponded Se ions quantification by traditional inductively coupled plasma mass spectrometer (ICP-MS). As displayed in Supplementary Figure S6, the Se concentration significantly increased in the first 2 h, while the tumor concentrated rate drastically reached a plateau at 6 h. Then it decreased from this timepoint on, after post-injection of hmSeO_2_@ICG-RGD at 96 h, insignificant Se element was appraised in tumor tissue. The ICP-MS data of tumor site is in accordance with NIR II fluorescent bio-imaging results, confirming the long tumor retention ability of hmSeO_2_@ICG-RGD. As the two primary organs of phagocyte-enriched RES, liver and spleen, enriched most nanoparticles. Only −2% of Se concentration in two organs can be found after 6 days of tail vein injection, indicating the safely metabolized behavior of this nanoagent.

*Ex vivo* fluorescent images were then obtained to assess biodistribution in the major organs and tumors resected at 72 h post-injection. hmSeO_2_@ICG-RGD nanoparticles mainly accumulated in tumor tissues, where levels were clearly higher than those in all other tissues in the ICG group, which had undetectable NIR II signals (Figures 5B, E). *In vivo* photothermal images were also collected using an IR camera following irradiation with an 808 nm laser after intravenous injection with hmSeO_2_@ICG-RGD and ICG into carcinoma bearing nude mice. The temperature clearly peaked at 24 h post-injection of hmSeO_2_@ICG-RGD with a negligible increase detected in the free ICG group (Figure 5C), verifying the accuracy NIR II fluorescent imaging for tumor targeting estimation. Collectively, our *in vivo* NIR II fluorescent and photothermal imaging data indicate that the hmSeO_2_@ICG-RGD nanoplatform has several advantages, including precise tumor targeting and long-term tumor accumulation, relative to free ICG, and could also act as an infrared thermograph nanoagent.

### 3.5 *In vivo* tumor eradication *via* synergistic therapy

**FIGURE 6:**
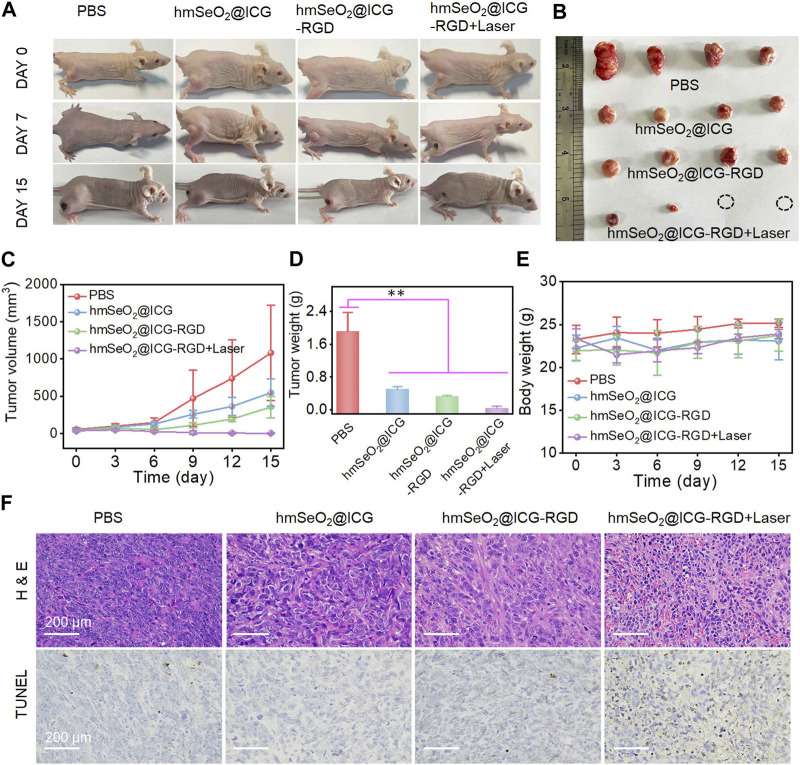
**(A)** Representative photographs of breast carcinoma-bearing BALB/c nude mice 0, 7 and 15 days after various treatments. **(B)** Representative resected tumors from mice in the various groups 15 days after treatment administration. **(C)** Tumor volumes in mice following various treatments. **(D)** Tumor weights after 15 days of treatment. **p < 0.01. **(E)** Body weights of mice in each treatment group. **(F)** H&E and TUNEL-staining photographs of tumor tissues resected from subcutaneous tumor-bearing mice after various treatments for 7 days.

In light of the observed effective tumor cell targeting using our nanoparticle system, we further assessed its tumor eradication efficacy *via* PTT and ROS-mediated oxidation effects in mammary carcinoma-bearing mice. First, animals were divided into four groups (n = 4 mice per group), which were administered with PBS, hmSeO_2_@ICG, hmSeO_2_@ICG-RGD, or hmSeO_2_@ICG-RGD + laser; in the last group, 808 nm laser irradiation was applied 24 h post-injection. Digital photographs of mice in the treatment groups were recorded on days 0, 7, and 15. Tumor volumes were also calculated in all groups during the tumor suppression period. As shown in Figures 6A, C, tumor growth was significantly suppressed in animals treated with our nanotherapeutic agent, including those in the ICG delivery (hmSeO_2_@ICG), tumor targeting (hmSeO_2_@ICG-RGD), and laser exposure (hmSeO_2_@ICG-RGD + laser) groups, relative to controls treated with PBS. Further, the sizes of four representative tumors dissected on day 15 were consistent with the observed tumor growth trends (Figure 6B). Partial tumor ablation was observed in the hmSeO_2_@ICG-RGD group, confirming the low efficiency of ROS-induced oxidation therapy. Notably, two tumors in the hmSeO_2_@ICG-RGD + laser group were completely ablated, in sharp contrast with those in the other three groups, and markedly lighter tumor weights were recorded in this group (Figure 6D). Overall, our analysis of tumor growth inhibition indicated that synergistic therapy mediated by hyperthermia improved ROS oxidation effects. H&E and TUNEL staining of tumor sections were also used to evaluate this synergistic therapeutic effect. As shown in Figure 6F, histopathological damage was accompanied by condensed tumor cell nuclei in the hmSeO_2_@ICG-RGD + laser group, while some tumor cell damage was also detected in tumors exposed to ROS generation alone (hmSeO_2_@ICG and hmSeO_2_@ICG-RGD). No significant variation in body weight was detected during the entire treatment period (Figure 6E). In striking contrast to the survival rate of PBS treated mice (35%), that of the hmSeO_2_@ICG-RGD + Laser administrated mice can prominently extend to 95% (Supplementary Figure S7). Further, mice in all treatment groups exhibited no visible organ damage by day 15, verifying that hmSeO_2_@ICG-RGD + laser treatment did no cause obvious systemic toxicity (Supplementary Figure S8). Overall, these results reveal that our nanoplatform successfully achieved tumor suppression, based on synergistic effects with laser ablation, and resulting from its excellent biocompatibility, high PTT efficacy, and considerable ROS generation ability.

## 4 Conclusion

In summary, here we describe the design of a multifunctional nanoplatform for tumor diagnosis and synergistic therapy based on hmSeO_2_ nanocarriers loaded with ICG and with RGD surface modification. This nanosystem precisely targets tumor cells and biodegrades into Se-based nanogranules in tumor microenvironments with weak acid pH values; Therefore, ICG is effectively released into tumor tissue facilitating NIR II fluorescence imaging. After tail vein administration of our nanoplatform into breast carcinoma-bearing mice, a significant high signal to background ratio was detected at 24 h post-injection, with extended tumor retention for up to 72 h post-injection. Further, our novel nanocarriers can generate ROS; in particular, hyperthermia from ICG accompanied by 808 nm laser irradiation facilitates ROS amplification. Furthermore, hmSeO_2_@ICG-RGD + laser treatment had no adverse side effects and showed high tumor suppression *via* PTT, as PTT enhances ROS generation mediating oxidative therapy. Overall, this study established an efficient strategy to realize simultaneous diagnosis *via* fluorescent imaging and elimination of malignant tumors *in vivo* in the clinic.

## Data Availability

The original contributions presented in the study are included in the article/Supplementary Material, further inquiries can be directed to the corresponding authors.
