# Corrigendum: Biodegradable hollowed mesoporous SeO_2_ nanoplatform loaded with indocyanine green for simultaneous NIR II fluorescence imaging and synergistic breast carcinoma therapy

**DOI:** 10.3389/fbioe.2023.1201751

**Published:** 2023-04-20

**Authors:** Tingwei Peng, Qing Liu, Hui Song, Conghui Zhang, Xue Wang, Ping Ru, Tianzhao Xu, Xinghui Liu

**Affiliations:** ^1^ Shanghai Gongli Hospital, Ningxia Medical University, Shanghai, China; ^2^ Department of Clinical Laboratory, Shanghai Gongli Hospital, The Second Military Medical University, Shanghai, China; ^3^ Department of Obstetrics, Shanghai East Hospital, School of Medicine, Tongji University, Shanghai, China; ^4^ Hospital Department, Shanghai University of Medicine and Health Sciences Affiliated to Zhoupu Hospital, Shanghai, China

**Keywords:** hollowed mesoporous SeO_2_, ICG precise delivery, NIR II fluorescent imaging, photothermal therapy, ROS mediated oxidative therapy

In the published article, there was an error in Figure 6A as published. The photograph of nude mouse is wrong in hmSeO_2_@ICG-RGD group. The corrected Figure 6 and its caption appear below.

**FIGURE 6:**
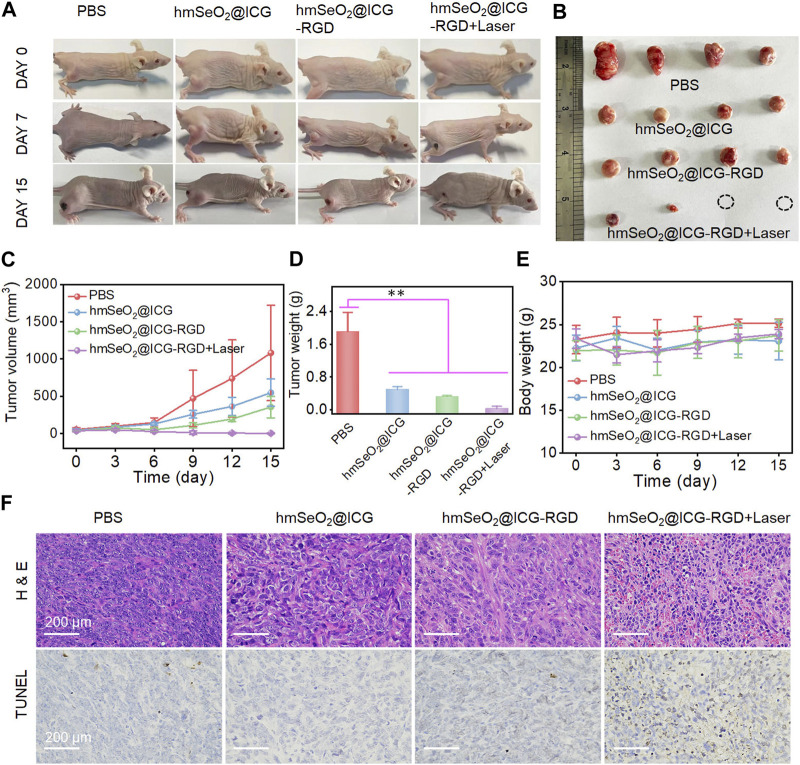
**(A)** Representative photographs of breast carcinoma-bearing BALB/c nude mice 0, 7 and 15 days after various treatments. **(B)** Representative resected tumors from mice in the various groups 15 days after treatment administration. **(C)** Tumor volumes in mice following various treatments. **(D)** Tumor weights after 15 days of treatment. **p < 0.01. **(E)** Body weights of mice in each treatment group. **(F)** H&E and TUNEL-staining photographs of tumor tissues resected from subcutaneous tumor-bearing mice after various treatments for 7 days.

The authors apologize for this error and state that this does not change the scientific conclusions of the article in any way. The original article has been updated.

