# Supplementary material for: Biodegradable hollowed mesoporous SeO2 nanoplatform loaded with indocyanine green for simultaneous NIR II fluorescence imaging and synergistic breast carcinoma therapy
Source: Front Bioeng Biotechnol. 2023 Mar 16;11:1151148. doi: 10.3389/fbioe.2023.1151148 (PMC10060888; doi:10.3389/fbioe.2023.1151148)
Supplement: Supplementary file 1 [file DataSheet1.docx]

**Biodegradable Hollowed Mesoporous SeO_2_ Nanoplatform loaded with Indocyanine Green for Simultaneous NIR II Fluorescence Imaging and Synergistic Breast Carcinoma Therapy**

**Tingwei Peng ^1#^, Qing Liu^2#^, Hui Song^2#^，Conghui Zhang^2^，Xue Wang^2^，Ping Ru^3*^ Tianzhao Xu ^4*^, Xinghui Liu^2*^**

^1^ Postgraduate training base in Shanghai Gongli Hospital, Ningxia medical university, Pudong New Area, Shanghai 200135, P.R. China.

^2^ Department of Clinical Laboratory, Shanghai Gongli Hospital, the Second Military Medical University, Pudong New Area, Shanghai, 200135, P.R. China.

^3^ Department of Obstetrics, Shanghai East Hospital, School of Medicine, Tongji University, Shanghai 200092, China.

^4^ Department of Hospital, Shanghai University of Medicine & Health Sciences affiliated Zhoupu Hospital, Pudong New District, Shanghai 201318, China.

*** Correspondence:**Xinghui Liu
[syliuxh@163.com](mailto:syliuxh@163.com)

Tianzhao Xu

[tianzhaoxufan@163.com](mailto:tianzhaoxufan@163.com)

Ping Ru

[ruping_rp@163.com](mailto:ruping_rp@163.com)

Keywords: Hollowed Mesoporous SeO_2_, ICG precise delivery, NIR II fluorescent imaging, Photothermal therapy, ROS mediated oxidative therapy

**Supplementary Experimental Section**

1. **Materials and characterization.**

***1.1 Materials.***

Hexadecyltrimethylammonium bromide (CTAB), Selenium tetrachloride (Se_2_Cl_2_), Hexamethyleneimine (C_6_H_12_N_4_) were purchased from Sigma-Aldrich. NaOH, cyclohexane and ethanol were obtained from Shanghai Chemical Co., Ltd. Ammonia aqueous solution (28 wt %), tetraethyl orthosilicate (TEOS), triethanolamine (TEA), decahydronaphthalene (98 %) were purchased from Aladdin Industrial Inc. All chemicals were used as received without further purification. ICG DSPE-PEG_2000_-NH_2_, 2',7'-Dichlorodihydrofluorescein diacetate, DAPI and RGD were purchased from Beyotime Biotechnology. Deionized (DI) water was used in all experiments.

- 1. ***Characterization.***

Transmission electron microscopy (TEM) measurements were carried out on a JEM 2100F microscope (Japan) operated at 200 kV. HRTEM and EDS mapping images were carried out in a Bruker Multimode 8, high-resolution transmission electron microscope operating at 200 Kv LEO1530VP SEM (Germany). SEM measurement was analyzed using Nanoscope V multimode atomic force microscope. The samples were first dispersed in ethanol and then collected by using copper grids covered with carbon films for measurements. UV-*vis*-NIR absorption spectra were measured on a Shimadz spectrophotometer (UV-3150) (Japan) with wavelength range of 300-1200 nm, unless otherwise specified, all spectra were collected under identical experimental conditions. Thermographic images were conducted by an IR camera (FOTRIC 225s). In vivo NIR II fluorescent images were obtained under a NIR-OPTICS Series III 900/1700 system (808 nm laser irradiation with a 1000 nm long filter pass).

**2. Photothermal conversion efficiency of hmSeO_2_@ICG-RGD**

***2.1 Photothermal conversion efficiency evaluation.***

The η value (photothermal conversion efficiency) of hmSeO_2_@ICG-RGD was determined as follows: 150 μg/mL of hmSeO_2_@ICG-RGD were added into the DI water and irradiated by 808 nm laser (1 W/cm^2^, 5 min). The temperature curve during laser on/off was measured and η can be apprised by the following two equation.

$\eta=\frac{hA\triangle T_{max}-Q_{s}}{I (1-{10}^{{-A}_{\lambda}})}$ (1)

$\tau=\frac{m_{D}c_{D}}{\mathrm{hA}}$ (2)

In formula (1), △*T*_max_ is the maximum temperature value from a stead state is 27.1°C for the hmSeO_2_@ICG-RGD (Figure 3E). Qs is the heat dissipation of water, which can be calculated as 0.0046. I is the power density of 808 nm laser 1 W/cm^2^ and A_λ_ is the absorbance of hmSeO_2_@ICG-RGD at 808 nm, which were recorded as 0.85. Then, the only hA value could be obtained from the following formula (2). In formula (2), m_D_ is the water mass, which is 0.2 g, c_D_ is the water capacity, which is 4.2 J/gK, and τ is the time constant for heat transfer which can be determined to be 121 s (Figure S1 in the revised supplementary file). According to this formula, hA is determined as 6.9×10^-3^ W/K. Therefore, η can be calculated as 39.87%.

**3. *In vitro* cellular targeting and cell killing of hmSeO_2_@ICG-RGD**

***3.1. Cell viability.***

All experiments were carried in 96-well plates. Cytotoxicity of hmSeO_2_@ICG-RGD was tested *via* CCK-8 kit assay. Briefly, the primary 4T1 isolated through enzymatic digestion were seeded into plate at 5×10^3^/well in 100 μL of 1640 (10% FBS, 100 units/mL of penicillin and 100 μg/mL of streptomycin), and incubated for 24 h. Then, mSeO_2_@ICG-RGD with various concentration was added followed by 12 h incubation. Lately, 10 μL CCK-8 in 1mL fresh culture medium was added to the cells, and after 2 h incubation, the absorbance of each well at wavelength of 450 nm was measured using a microplate reader. Data were presented as mean ± SD (n = 3). Meanwhile, cell viability of mSeO_2_@ICG-RGD toward normal cell line, HUVEC was carried out at same procedure.

***3.2 In vitro cell killing efficiency evaluation.***

Cell killing efficacy was evaluated after NIR laser irradiation. Briefly, the primary 4T1 tumor cells were isolated by enzymatic digestion and then seeded into 96 well plate (5×10^3^/well) in 100 μL of fresh 1640 medium (10% FBS, 100 units/mL of penicillin, 100 μg/mL of streptomycin) for 24 h incubation. Then, mSeO_2_@ICG-RGD with various concentrations for another 12 h incubation. Then cells were irradiated by 808 nm laser (1.0 W/cm^2^, 5 min), then live cell percentage was detected by CCK-8 assay.

***3.3 CLSM images of cellular uptake.***

The cellular uptake of ICG, mSeO_2_@ICG and mSeO_2_@ICG-RGD was investigated and imaged by a confocal laser scanning microscopy (CLSM). Briefly, 4T1 breast tumor cells were firstly seeded into 6-well plates at a concentration of 1×10^5^ per well with 1 mL 1640 fresh medium (10% FBS, 100 units/mL of penicillin, 100 μg/mL of streptomycin) for 24 h incubation. After the treatment with free ICG, mSeO_2_@ICG, mSeO_2_@ICG-RGD at the same concentration of ICG (5 μg/mL), the 4T1 breast cancer cells were incubated for 12 hours. Then, all the cell samples were washed by PBS for 3 times and 1 μg /mL DAPI was subsequently used to stain cellular nuclei for 0.5 h before CLSM observation.

***3.4 Live/Dead cell discrimination and cell apoptosis analysis***

Calcein-AM/PI experiments was carried out to evaluate the *in vitro* anti-tumor capability and mechanism. 4T1 tumor cells seeded into 6-well plate at the density as 1×10^5^per well with 1 mL of 1640 fresh media (10% FBS, 100 units/mL of penicillin, 100 μg/mL of streptomycin) for 24 h co-culture then the tumor cells were administrated by incubation of various formulations: PBS, ICG, mSeO_2_@ICG-RGD and mSeO_2_@ICG-RGD+laser. After laser irradiation at 12 h incubation (1.0 W/cm^2^, 5 min), all cells were stained by Calcein-AM/PI and live/dead cells were visualized by CLSM. Cell apoptosis studies were also performed. Cell samples were prepared as the sample procedure as live/dead discrimination, except for Anneix V/PI stanning followed by enzymatic digestion. The percentage of cellular apoptosis was further estimated by a flow cytometer.

**Supplementary Figures**


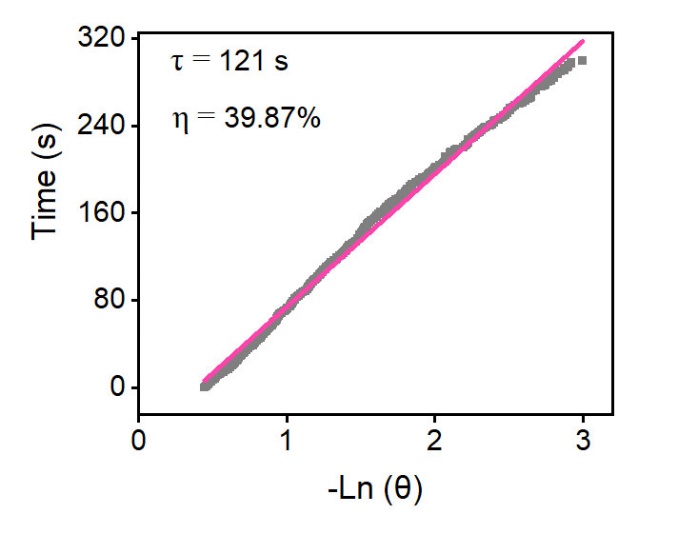


**Figure S1.** Cooling time versus –Ln (θ) line of hmSeO_2_@ICG-RGD.


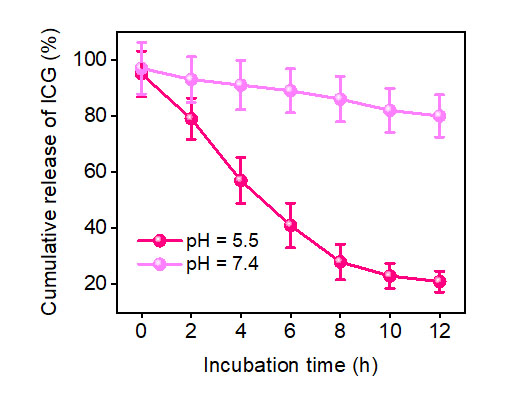


**Figure S2.** ICG release curves of hmSeO_2_@ICG-RGD after immersed with pH=5.5 or pH=7.4 buffer for various hours.


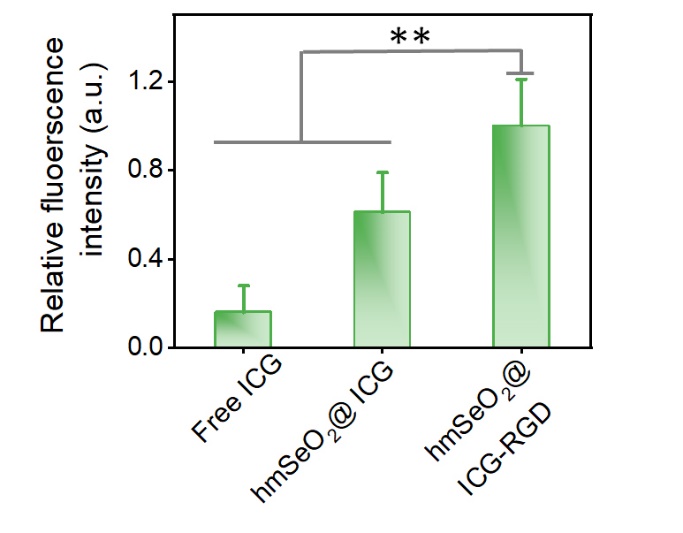


**Figure S3.** Quantatitively relative fluorescence intensity in Figure 4A. **p < 0.01.


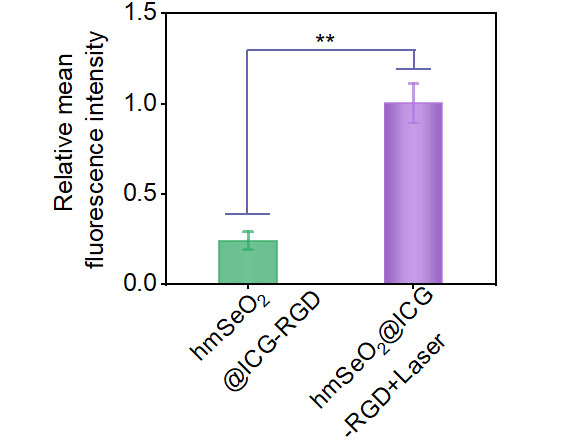


**Figure S4.** Relative mean intracellular ROS fluorescent intensity of hmSeO_2_@ICG-RGD and hmSeO_2_@ICG-RGD+Laser, respectively. **p < 0.01.


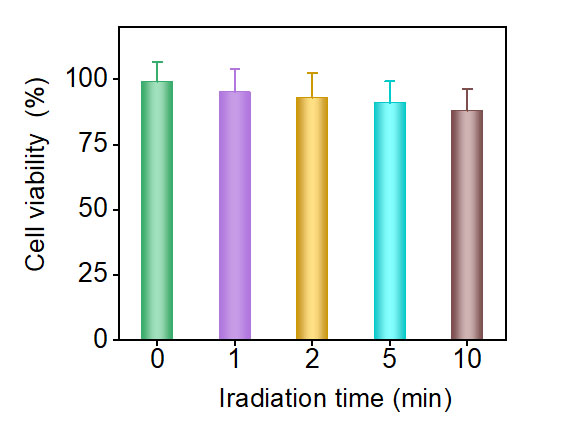


**Figure S5**. 4T1 cell viability investigation after 808 nm laser (1W/cm^2^) irradiation for various minutes.


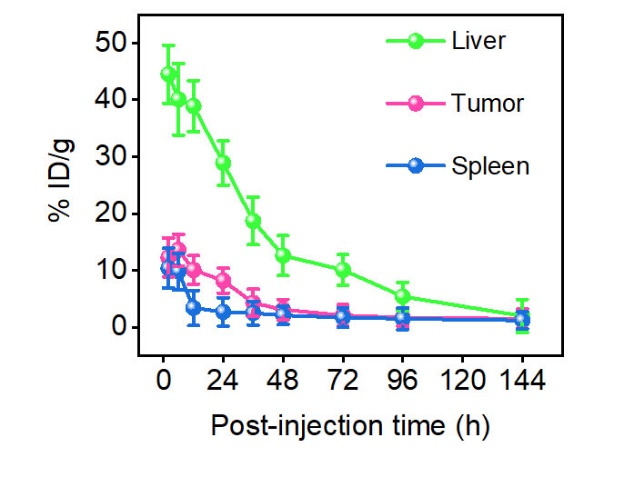


**Figure S6.** Se ions in liver, tumor and spleen collected after caudal injection of hmSeO_2_@ICG-RGD at various hours.


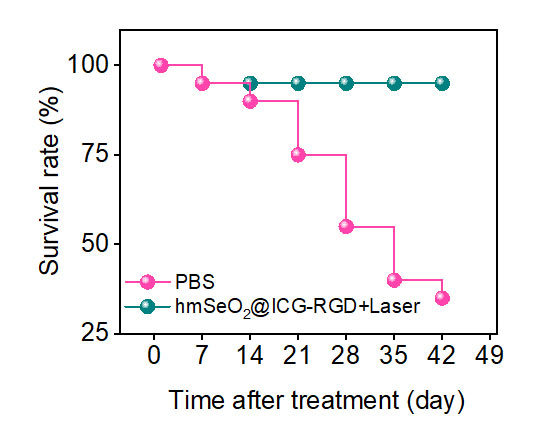


**Figure S7**. Survival rate of tumor bearing mice after 15 days treatment of hmSeO_2_@ICG-RGD + Laser or PBS.


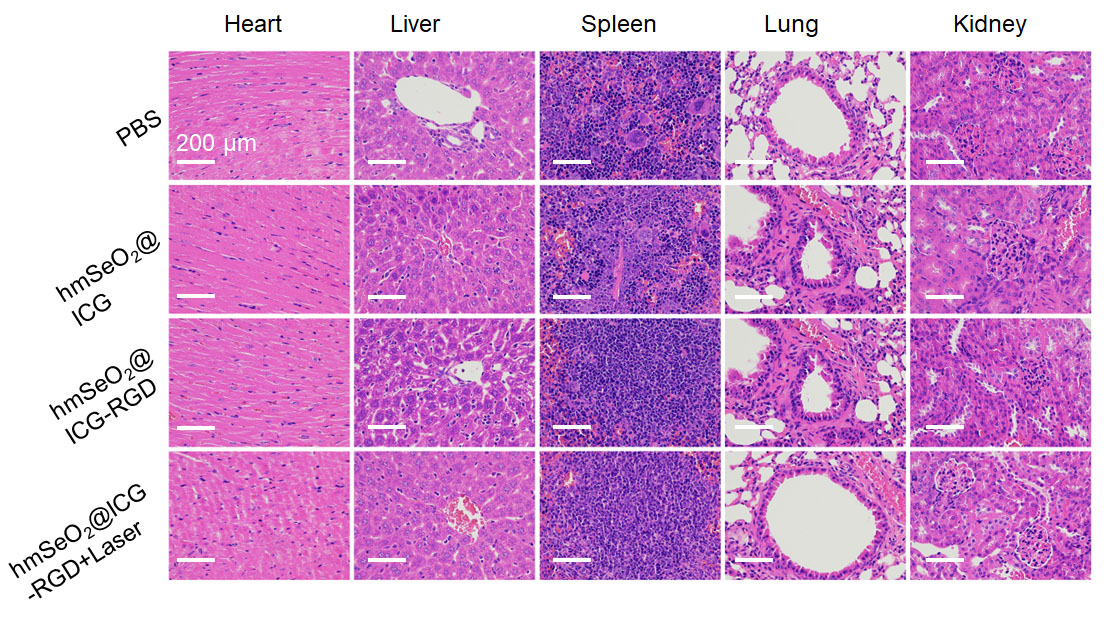


**Figure S8.** H&E staining photographs of main organs dissected from breast-carcinoma bearing Balb/c nude mice after 15 days treatments of PBS, hmSeO_2_@ICG, hmSeO_2_@ICG-RGD and hmSeO_2_@ICG-RGD+laser.
